# Prognostic significance of the digit ratio after hormone therapy for prostate cancer: a prospective multicenter study

**DOI:** 10.1038/s41598-017-05638-w

**Published:** 2017-07-12

**Authors:** Guanjian Li, Ke Sun, Jie Guo, Shixing Li, Bo Li, Jing Cao, Pengfei Lu, Jiajia Yang, Ying Zhang, Xin Yang, Le Gao, Yi He, Tao Cui, Bin Ma

**Affiliations:** 1 The second Affiliated Hospital Of Xinjiang Medical University, Xinjiang, China; 2 School of Public Health, Peking University, Beijing, China; 3 China-Japan Union Hospital of Jilin University, Jilin, China; 4 Norman Bethune Health Science Center of Jilin University, Jilin, China; 5 Shandong Provincial Hospital, Shandong, China; 6 Cheeloo College of Medicine, Shandong University, Shandong, China; 7 XinJiang Medical University, Xinjiang, China; 8 School of Public Health, Xinjiang Medical University, Xinjiang, China

## Abstract

The digit ratio has been used as a retrospective noninvasive biomarker to investigate the putative effects of prenatal exposure to androgens. In recent years, many scholars have paid attention to the association between 2D:4D (the second and fourth digits) and prostatic cancer. This study explored the prognostic significance of digit ratio in prostate cancer patients. We reviewed the progressive status and survival of 382 prostate cancer patients who had received hormone therapy at our institutions. Survival of clinicopathological variables analyzed as categorical variables were determined by the log-rank test. According to Cox’s proportional hazards analysis, R2D:4D, L2D:4D, PSA at 6 month,bone metastasis were significant independent factors for prostate cancer. The risk of any progression of prostate cancer similarly depressed with increasing 2D:4D, for any progression (R2D:4D HR = 0.71, p = 0.003; L2D:4D HR = 0.67, p = 0.001), for cancer-specific death (R2D:4D HR = 0.67, p = 0.025; L2D:4D HR = 0.74, p = 0.036). Digit ratio may not only have predictive value in risk but also prognosis of prostatic cancer. This finding suggests that low 2D:4D can be used as prognostic factors to identify patients with a poor prognosis. These patients may benefit from more aggressive management.

## Introduction

Prostate cancer is one of the most common malignant tumors in men, with a reported incidence of 165.8 per 100, 000^1^. In China, it is known that prostate cancer is a serious public health problem. For now, this cancer is the second-leading cause of cancer deaths in men, after lung and bronchial cancer, according to the National Cancer Prevention Office. Although exact etiology of prostate cancer is currently unknown, it has been correlated to factors such as age, race, familial history, dietary habits and hormone exposure^2^.

The ratio between the second and fourth digits (2D:4D), also known as digit ratio is a proxy marker for the prenatal influence of sexual hormones - mainly testosterone and estrogen^3–5^. In mice, nineteen genes have been identified to influence in digit ratio establishment^6^, and the HOX, androgen receptor (AR) and LIN28B have been speculated to reflect on digit ratio in humans^7, 8^. These genes are correlated with prenatal testosterone (PT) or prenatal estrogen (PE), being up or down regulated by the exposure to these hormones. So digit ratio can be thought of as a candidate marker for the action of these genes, and, subsequently for it used as a predictor of hormone-related disease susceptibility^9–12^.

In 2002, Manning *et al*. started to focus on the possible links between 2D:4D and reproductive cancers (prostate, breast, ovary, uterus), they suggested that low 2D:4D may be negatively related to high PSA and high risk of prostate cancer^13^. Recently, prostate cancer is the neoplasm most connected to 2D:4D, which has been studied by several ethnic groups such as American, Australian, Brazilian, British, Indian, Korean, and Spanish^9, 14–20^. These groups have evaluated the influence of digit ratio over the prevalence and severity of prostate cancer, as well as prostatic specific antigen (PSA) and Gleason scores.

However, no correlative literature has investigated the association between digit ratio and survival in prostate cancer patients, whether digit ratio can be used to predict the progressive status and survival has not been reported as far as we know.

Based on such considerations, this study was undertaken to explore the prognostic significance of digit ratio in prostate cancer patients who had received hormone therapy at our institutions.

## Methods

### Participants

This study was performed in three large academic medical centers in china. Patients with prostate cancer were recruited from the oncology and urology department of The Second Affiliated Hospital Of XinJiang Medical University, Shandong Provincial Hospital, China-Japan Union Hospital of Jilin University, and met the following inclusion criteria: having a diagnosis of prostate adenocarcinoma and undergoing whole-course endocrine therapy at our institution.

The study population consisted initially of 926 patients who underwent endocrine therapy from march 2011 to march 2014 at our institutions. Patients were excluded for the following reasons: History of related radiation or surgery (n = 435); Histologic slides, Gleason score, PSA data or digit ratio values not available (n = 25); Not right-handed, skeletal dysplasias, or who had suffered from any kind of injuries to the fingers (n = 43); Lack of research authorization (n = 41). Nevertheless, sedatives or analgesic medication, limited operations to remove lower urinary tract obstructions (cystostomy or catheterization) were permitted.

The final study group consisted of 382 prostate cancer patients who were diagnosed pathologically at our institutions from march 2011 to march 2014. Among these participants, 337 patients were treated with endocrine therapy, using medical castration (using a luteinizing hormone-releasing hormone analogue) with antiandrogens, and the remaining 45 patients were treated with simple medical castration.

Our study was approved by The Ethics Committee of The XinJiang Medical University and all of the relevant hospitals. Informed consent was provided according to the Declaration of Helsinki and all of the patients’ guardians informed written consent. We confirming that all experiments were performed in accordance with relevant guidelines and regulations that promote respect for all human subjects and protect their health and rights.

### Follow-up information

The median time of follow-up and the follow-up range were 45.0 months and 1–72 months, respectively.

All these patients were followed every month for the first 3 months and once every 3 months thereafter. Serum PSA levels, complete blood cell counts, blood chemistry studies, rectal examinations were recorded and analyzed. Serum levels of PSA were assayed by CanAg PSA ElA (CanAg Diagnostics AB). Meanwhile, chest x-rays and radionuclide bone scans (injection of 25 mCi Tc-99m methylene diphosphonate) were performed every 6 months. If the patient complained of subjective symptoms, computerized tomography of the abdomen and pelvis or abdominal ultrasonography was also performed to measure the disease’s progression. The TNM classification system was used for staging and the Gleason system was used for grading.

Any progression of the prostate cancer was defined as: (1) Progression to hormone refractory prostate cancer, when PSA increased after hormone treatment twice in succession and continued to increase even after suspension of flutamide; (2) Local recurrence, cancer on biopsy of prostatic bed without evidence of systemic recurrence. (3) Systemic progression, involved demonstrable metastatic deposits on biopsies, abdominal CT scan or bone scan. Prostate cancer death was defined as death in any patient with metastasis that showed any progression following hormonal therapy.

### Digit measurement

**Figure 1:**
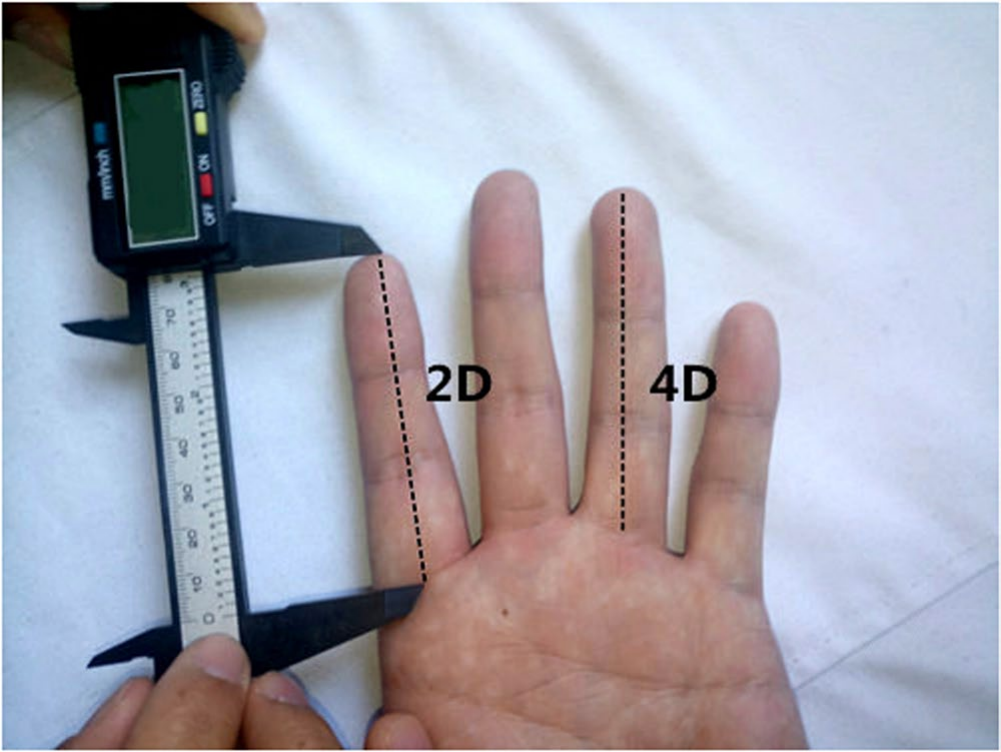
Measurement of digit length using vernier calipers.

Figure 1 shows how to measure the digit length. The 2D and 4D were measured by unified specially-trained investigators with a standard vernier caliper (recorded to 0.01 mm) from the basal palmar crease of the finger to the tip. This measurement has been previously reported providing a high degree of repeatability^21, 22^. When two creases were visible at the base of the digit, the crease proximal to the palm was chosen. The measurers had no information about the progression and therapy about the patients. All measurements were made twice by two investigators and the mean value was adopted. We got the 2D:4D values at the beginning of the treatment mostly. The intraclass correlation coefficient (ICC) of two repeated measures of digit ratio by a single investigator was 0.918. This direct measurement method is probably better, as indirect finger measurements (e.g. from photocopies or scans) may lead to distortions that result in consistently lower 2D:4D values^23, 24^.

### Statistical analysis

For the analysis of the results, the patients were divided into groups according to 2D:4D (<0.950 versus ≥0.950), pathological Gleason score (<6 or 7 versus 8–10), initial PSA (<20, 20–99.9, 100–1000, >1000), PSA at 3 month (<4 versus ≥4), PSA at 6 month (<4 versus ≥4), bone metastasis (yes versus no), lung metastasis (yes versus no), lymph node metastasis (yes versus no), therapy (monotherapy versus combined) and pathological tumor stage (TX, T0, T1 or T2 or T3 versus T4).

All statistical analyses were performed by using the SPSS software (SPSS Inc., Chicago, Illinois) with a significance level of 0.05.

Variables of the different groups were compared using Student’s t test or Fisher’s exact test. Survival curves were constructed by the Kaplan-Meier method, survival of clinicopathological variables analyzed as categorical variables were determined by the log-rank test. Multivariate analysis was then performed according to the Cox proportional hazards regression model to determine the significant factor in predicting disease outcome. To obtain a multivariate model with the maximum precision of the important variables, a stepwise selection procedure was used.

## Results

### Follow up results

Patient characteristics of the study are summarized in Table 1, all of the participants were Chinese Han patients.

**Table 1:** Patient characteristics.

Demographics	
No. assessable patients	382
Mean age (range; s. d.)	65.4 (34–89; 9.4)
Median followup (range)	45.0 (1–72)
Mean Gleason score (s. d.)	7.30 (1.93)
Mean initial PSA (s. d.)	362.69 (543.27)
No. bone metastasis (%)	248 (64.9)
No. lymph node metastasis (%)	115 (30.1)
No. pulmonary metastasis (%)	21 (5.5)
No. therapy (%)	
Monotherapy (chemical castration)	45 (11.8)
Combined androgen blockade	337 (88.2)
R2D:4D (s. d.)	0.949 (0.041)
L2D:4D (s. d.)	0.955 (0.045)

This study did not find a relationship between Gleason score and 2D:4D, but encountered significant negative correlation between 2D:4D and PSA at 3 months, 6 months after treatment (Table 2).

**Table 2:** Relationship between clinicopathological factors and 2D:4D values.

	No. (%)	R2D:4D (s. d.)	P value	L2D:4D (s. d.)	P value
Overall	382(100)	0.949 (0.041)		0.955 (0.045)	
Age/yr			0.132		0.117
<65	68(17.8)	0.953 (0.049)		0.955 (0.045)	
65–75	217(56.8)	0.949 (0.046)		0.948 (0.041)	
>75	97(25.4)	0.948 (0.044)		0.957 (0.052)	
Pathological Gleason score			0.294		0.355
<6	73(19.1)	0.952 (0.038)		0.955 (0.051)	
7	162(42.4)	0.949 (0.055)		0.957 (0.055)	
8–10	147(38.5)	0.946 (0.050)		0.948(0.045)	
Initial PSA (ng./ml.)			0.420		0.324
<20	57(14.9)	0.944 (0.041)		0.960 (0.044)	
20–99.9	143(37.4)	0.950 (0.046)		0.951 (0.052)	
100–1000	158(41.4)	0.951 (0.040)		0.957 (0.053)	
>1000	24(6.3)	0.947 (0.047)		0.955 (0.040)	
PSA at 3 month (ng./ml.)					
<4	167(45.3)	0.955 (0.060)	0.039	0.958 (0.055)	0.046
≥4	202(54.7)	0.944 (0.052)		0.951 (0.043)	
PSA at 6 month (ng./ml.)					
<4	195(57.0)	0.957 (0.056)	0.001	0.963 (0.045)	0.001
≥4	147(43.0)	0.939 (0.063)		0.945 (0.055)	
Bone metastasis			0.524		0.386
yes	248 (64.9)	0.950 (0.036)		0.957 (0.053)	
no	134	0.948 (0.037)		0.952 (0.052)	
Lymph node metastasis			0.072		0.129
yes	115 (30.1)	0.953 (0.056)		0.956 (0.051)	
no	267 (69.9)	0.944 (0.053)		0.955 (0.044)	
Lung metastasis			0.861		0.520
yes	21 (5.5)	0.945(0.060)		0.960 (0.057)	
no	361 (94.5)	0.950 (0.039)		0.954(0.042)	
Pathological tumor stage			0.202		0.334
T_X_, T_0_, T_1_	32 (5.8)	0.953 (0.036)		0.960 (0.039)	
T_2_	129 (33.8)	0.949 (0.055)		0.953 (0.046)	
T_3_	122 (34.6)	0.946 (0.057)		0.958 (0.045)	
T_4_	99 (25.9)	0.948 (0.048)		0.954 (0.039)	
No. therapy (%)			0.290		0.481
Monotherapy3sd	45 (11.8)	0.962 (0.044)		0.952 (0.060)	
combined	337 (88.2)	0.949 (0.040)		0.956 (0.038)	

Among 382 patients, 281 (73.6%) patients had disease progression (as defined above), 117 (30.6%) died of prostate carcinoma, 11 (2.9%) were non-cancer related deaths, and 20 (5.2%) were excluded from analysis due to loss to follow-up after treatment.

### Univariate and multivariate analysis

Individual parameters were evaluated using univariate analysis for possible correlation with any progression of prostate cancer, R2D:4D, L2D:4D, initial PSA, PSA at 6 month after treatment, bone metastasis, lymph node metastasis influenced the progression (Table 3). However, by multivariate analysis, stepwise inclusion of variables in the Cox’s proportional hazards model showed that the significant prognostic factors were R2D:4D, L2D:4D, initial PSA, PSA at 6 month, bone metastasis were significant independent factors (Table 4).

**Table 3:** Results of univariate analysis of prognostic factors for time to any progression free survival and cancer sepecific survival (log-rank).

Variable	No. (%)	Median progression free survival time	P value	Median cancer sepecific survival time	P value
R2D:4D			0.015		0.036
<0.950	218 (57.1)	23			
≥0.950	164 (42.9)	29			
L2D:4D					
<0.950	179 (46.9)	22	0.001		0.004
≥0.950	203 (53.1)	33			
Pathological Gleason score			0.246		0.303
<6	73 (19.1)	30			
7	162 (42.4)	26			
8–10	147 (38.5)	22			
Initial PSA (ng./ml.)					
<20	57 (14.9)	46	0.001		0.001
20–99.9	143 (37.4)	27			
100–1000	158 (41.4)	25			
>1000	24 (6.3)	22		42	
PSA at 3 month (ng./ml.)					
<4	167 (45.3)	29	0.159		0.133
≥4	202 (54.7)	27			
PSA at 6 month (ng./ml.)			0.011		0.001
<4	195 (57.0)	30			
≥4	147 (43.0)	23		60	
Bone metastasis			0.007		0.001
yes	248 (64.9)	19		47	
no	134	34			
Lymph node metastasis			0.025		0.041
yes	115 (30.1)	23			
no	267 (69.9)	29			
Lung metastasis			0.472		0.280
yes	21 (5.5)	22			
no	361 (94.5)	28			
Pathological tumor stage			0.297		0.234
T_X_, T_0_, T_1_	22 (5.8)	36			
T_2_	129 (33.8)	29			
T_3_	132 (34.6)	26			
T_4_	99 (25.9)	24		45	
No. therapy (%)			0.122		0.083
monotherapy	45 (11.8)	26			
combined	337 (88.2)	27

**Table 4:** Cox proportional hazards model for predicting any cancer progression (Hazards ratio estimates and 95% CI). All estimates adjusted.

Variables	HR*	P value	95% CI
R2D:4D	0.71	0.003	0.57–0.91
L2D:4D	0.67	0.001	0.54–0.86
Initial PSA (ng./ml.)			
<20	1.00		
20–99.9	1.35	0.067	0.95–2.23
100–1000	2.47	0.018	1.16–4.22
>1000	2.57	0.001	1.03–5.39
PSA at 6 mos. (ng./ml.)	1.68	0.012	0.81–3.38
Bone metastasis	2.95	0.035	0.94–5.20

*Variables were used as categorical, as in Table 2.

According to univariate analysis, R2D:4D, L2D:4D, initial PSA, PSA at 6 month after treatment, bone metastasis and lymph node metastasis affected the cancer-specific death (Table 3). However, Cox’s proportional hazards analysis using a stepwise inclusion of variables demonstrated that R2D:4D, L2D:4D, PSA at 6 month, bone metastasis were significant independent factors (Table 5).

**Table 5:** Cox proportional hazards model for prostate carcinoma death (Hazards ratio estimates and 95% CI). All estimates adjusted.

Variables	HR*	P value	95% CI
R2D:4D	0.67	0.025	0.51–0.97
L2D:4D	0.74	0.036	0.51–1.06
PSA at 6 mos. (ng./ml.)	2.35	0.004	1.07–4.58
Bone metastasis	1.87	0.013	0.98–3.40

*Variables were used as categorical, as in Table 2.

### The survival curves

**Figure 2:**
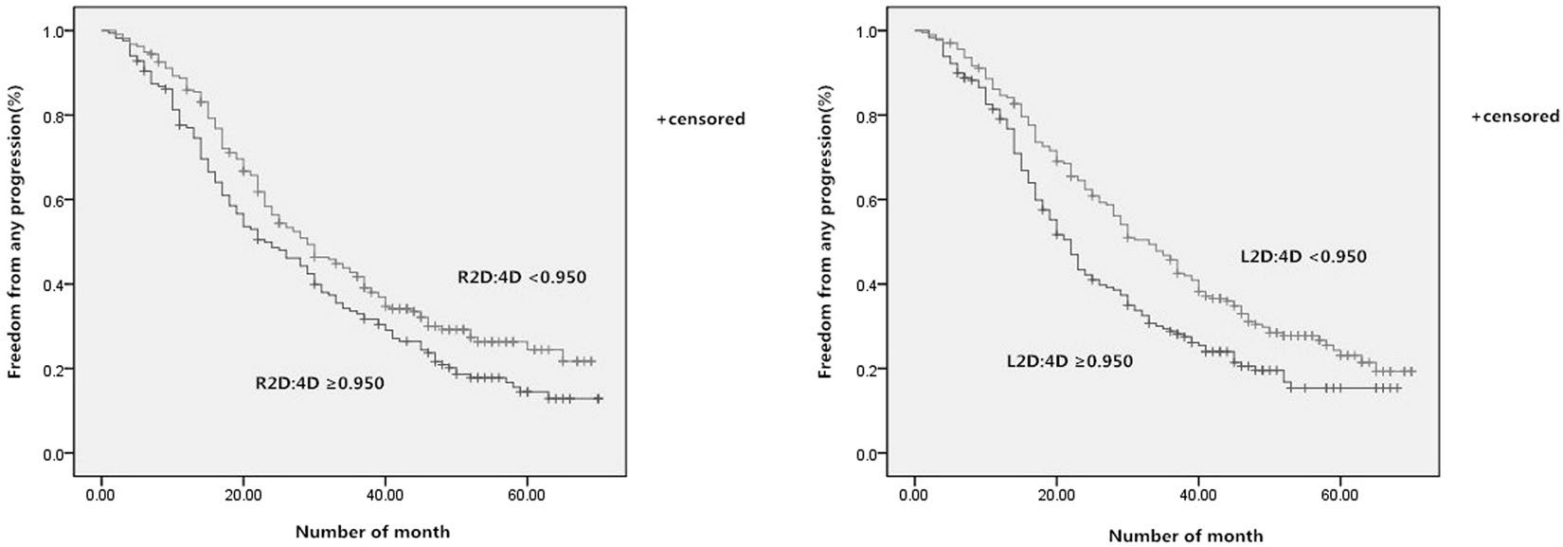
Kaplan–Meier curves for any progression free survival, according to the R2D:4D and L2D:4D (<0.950 versus ≥0.950).

**Figure 3:**
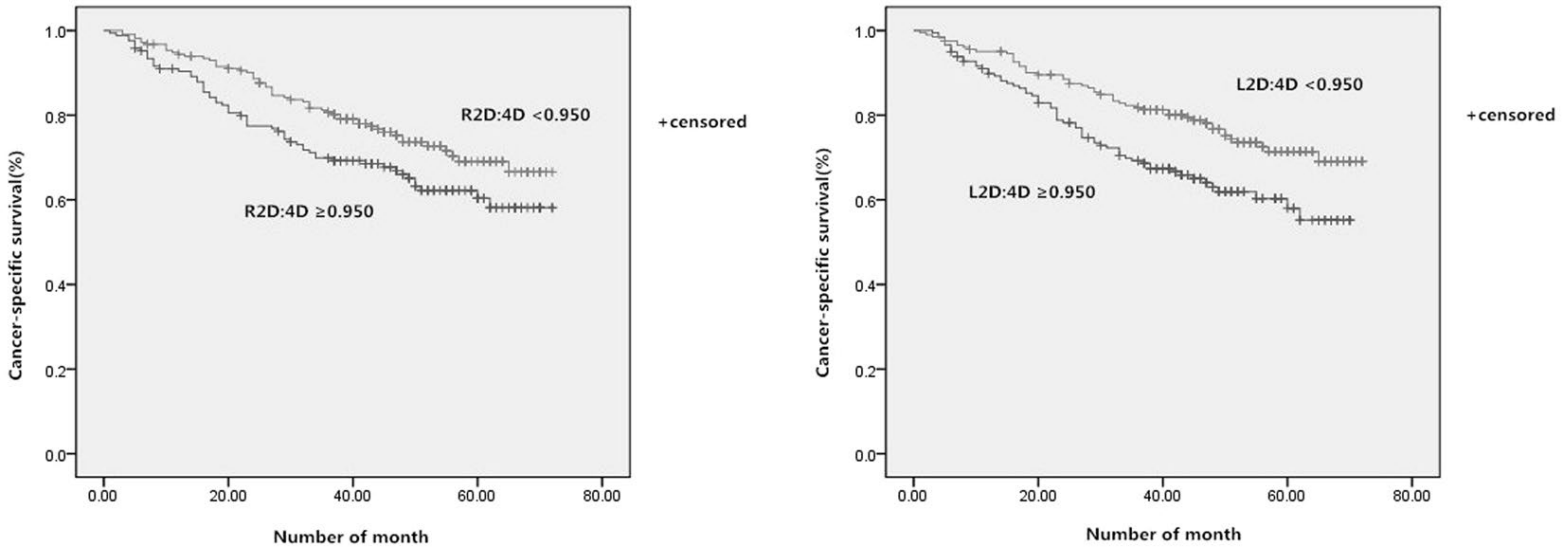
Kaplan–Meier curves for cancer specific survival, according to the R2D:4D and L2D:4D (<0.950 versus ≥0.950).

The risk of any progression of prostate cancer similarly depressed with increasing 2D:4D (Fig. 2). While patients with a higher 2D:4D were at nearly 0.7 depressed risk for any progression (R2D:4D HR 0.71, 95% CI 0.57–0.91, p = 0.003; L2D:4D HR 0.67, 95% CI 0.54–0.86, p = 0.001). Similarly, for cancer-specific death (Fig. 3), higher 2D:4D depressed the risk as well (R2D:4D HR 0.67, 95% CI 0.51–0.97, p = 0.025; L2D:4D HR 0.74, 95% CI 0.51–1.06, p = 0.036).

## Discussion

Several converging lines of evidence suggest that prenatal hormone exposure is associated with digit ratio, and much of relevant evidence has been previously reviewed^25^. A direct evidence, Lutchmaya *et al*. reported that L2D:4D is negatively correlated with the ratio of testosterone to oestradiol measured by amniocentesis in the second trimester^3^. HOX genes regulate the embryogenesis, morphogenesis and cell differentiation of human, including the genitals and digits, and they have been suggested to be deregulated in malignant tumors^26, 27^. In primary prostate cancer cells, HOX8 or MSX2 gene expressions are greatly reduced, followed by a gradual increase in more aggressive prostate cancer cell lines^28^. Despite the controversy and criticism^29^, it has been postulated that digit ratio was associated with the CAG repeats number in the AR (androgen receptors) genes^30^. It is generally known that testosterone and AR play crucial roles in prostate growth and the progress of prostate cancer. The short CAG repeats of AR have been reported to be associated with the pathogenesis of prostate cancer^31, 32^. Furthermore, the polymorphisms of AR gene may occur in advanced prostate cancer, which became resistant to hormonal treatment^33, 34^.

Recently, many experts and scholars had a strong interest in the relation between 2D:4D and prostate. In a case-control study, compared with low 2D:4D men, abnormal development (persistence of mid-line cyst formation) of the prostate was more common in men with high 2D:4D. This suggests that 2D:4D may relate to the normal development of prostate^35^. In another convincing study, the researchers found that the digit ratio is a predictor of the response to dutasteride (one inhibitor of the action of 5α-reductase) treatment^36^. This suggests that the 2D:4D may be linked to the function of the prostate. In tumor research studies, prostate cancer is the cancer most connected to 2D:4D, which is considered to develop as a result of hormonal carcinogenesis. Whereby hormones promote cell division and cell proliferation, and the accumulation of genetic mutations that arise during the process of cell division eventually result in a malignant phenotype^37^.

In 2010, a significant study to evaluate the correlations between 2D:4D and prostate cancer found that individuals with a higher risk of prostate biopsy possessing high levels of PSA presented lower 2D:4D values^20^. Then, in a major cohort study in the United Kingdom, researchers have proposed that 2D:4D may be a simple marker of prostate cancer risk, with length of 2D greater than 4D suggestive of lower risk^9^. Another investigation in 2012, the Korean researchers found an association between 2D:4D, prostate cancer volume and Gleason score after investigating 770 men with lower urinary tract symptoms^38^. However, in 2013, one US study found lower right-hand digit ratios for African-Americans with prostate cancer in comparison to Caucasians, but no significant differences for Gleason scores^14^. Similarly, in our study, we found no correlation between 2D:4D and Gleason scores either. Such differences may reside in the different evaluation of digit ratios, technique use, data processing mode and the different backgrounds of the samples (patients condition and prognosis, ethnic). In short, prostate cancer is a kind of multifactorial disease and influenced by both ethnic, environmental and genetic factors, larger multi-center prospective study may be necessary for further research.

In 2011, when we started this research, we measured the digit ratio and collected the relevant data of prostate cancer patients to examine whether 2D:4D is associated with prostate cancer risk. At that time, more and more attentions were paid to this subject in academia, and many major studies carried out. Then, we noted that all of these experiments restricted to investigations on the relationship between 2D:4D and risk (prevalence or severity) of prostate cancer, whether associations exist between 2D:4D and the disease prognosis was never mentioned. So, based on previous researches, we turned our attention to the prognostic significance of digit ratio in prostate cancer patients. During an average follow-up of 45.0 months, we found that digit ratio may not only have predictive value in risk but also prognosis of prostatic cancer finally.

Due to limited practical condition and research competence, our study has several potential limitations. Prostate cancer is indolent tumor that has long survival, even with metastases^39^. In our study, with relatively short-term following up, the number of outcome events was limited. Therefore, our results should be considered preliminary. So, longer-term studies are desperately needed to catch more significant differences in the progression.

Actually, the use of 2D:4D for outcome prediction may be not as straightforward as the result suggest. The main reason is that ethnic groups differ in mean 2D:4D. In a meta-analysis on Chinese 2D:4D, of the results of 28 studies (19,093 participants), the left 2D:4D was 0.951 in Chinese men, Q (26) = 658.61, P < 0.001, and the right 2D:4D was 0.948, Q (28) = 753.56, P < 0.001^40^. After analyzing the data of relevant research, it was found that the 2D:4D in Chinese individuals (Han ethnicity) was found to be higher than in Black populations but lower than in Caucasians and Hispanics^41–43^. These ethnic differences in 2D:4D further indicate that genetic factors likely affect the 2D:4D. Furthermore, the diagnosis, therapy and prognosis of prostatic cancer show significant differences in different countries and nationality^44, 45^.For instance, the screening of prostate cancer in China is not as widespread as in Western countries, and the age-adjusted incidence and mortality rates of prostate cancer in Asia are much lower than Europe and the United States. In conclusion, due to all of the participants are Chinese Han in our study, the conclusion may be specific to the particular ethnic and regional composition of our study sample.

Since Huggins and Hodges found the beneficial effect of androgen ablation on metastatic prostate cancer in 1941^46^. androgen deprivation therapy, specifically medical castration, has been the first line treatment for advanced prostate cancer. And, there have been many experiments to study diverse prognostic factors for prostate cancer^47–49^. In our study, the results indicate that digit ratio may be associated with the prognosis of patients with prostate cancer, and 2D:4D can be used as prognostic factors to identify patients with a poor prognosis, who may benefit from more aggressive management. At present, detailed interpretation of this result is difficult due to the fact that we can only provide an indication that prenatal androgen exposure might be associated with the prognosis of prostatic cancer, the biochemical pathway or molecular mechanism cannot be explained systematically. Here we offer two possible explanations: 1) differences in fetal exposure to androgens or the sensitivity of AR may be related to the appearance and proliferation of the receptor-negative tumour cells, and 2) by other possible mechanism of gene regulation, prenatal androgen exposure may be associated with the proliferation, invasion, migration, apoptosis and multi drug resistance of tumor cells.

In conclusion, despite this current limitation, 2D:4D remains a simple, easy to measure marker of prenatal androgen exposure which may not only have predictive value in risk but also prognosis of prostatic cancer.
